# Functional diversification and molecular mechanisms of *FLOWERING LOCUS T*/*TERMINAL FLOWER 1* family genes in horticultural plants

**DOI:** 10.1186/s43897-022-00039-8

**Published:** 2022-08-16

**Authors:** Shuang Wang, Yiman Yang, Fadi Chen, Jiafu Jiang

**Affiliations:** State Key Laboratory of Crop Genetics and Germplasm Enhancement, Key Laboratory of Landscaping, Ministry of Agriculture and Rural Affairs, Key Laboratory of Biology of Ornamental Plants in East China, National Forestry and Grassland Administration, College of Horticulture, Nanjing Agricultural University, Nanjing, 210095 China

**Keywords:** FLOWERING LOCUS T (FT), TERMINAL FLOWER 1 (TFL1), Flowering, Functional evolution, Horticultural plant

## Abstract

Flowering is an important process in higher plants and is regulated by a variety of factors, including light, temperature, and phytohormones. Flowering restriction has a considerable impact on the commodity value and production cost of many horticultural crops. In *Arabidopsis*, the *FT/TFL1* gene family has been shown to integrate signals from various flowering pathways and to play a key role in the transition from flower production to seed development. Studies in several plant species of the *FT/TFL1* gene family have revealed it harbors functional diversity in the regulation of flowering. Here, we review the functional evolution of the *FT/TFL1* gene family in horticulture plants and its unique regulatory mechanisms; in addition, the *FT/TFL1* family of genes as an important potential breeding target is explored.

## Introduction

Flowering is an important stage in the life history of higher plants that includes the processes of flower bud differentiation, development, and the opening of flowers (Parmar et al. 2017; Xu et al. 2019). An optimal flowering is of great significance for plants to complete their life cycle under suitable environmental conditions (Su et al. 2019). Horticultural plants are critical components of agricultural production; they include fruits, flowers, vegetables, spices, medicinal, and aromatic plants (Karkute et al. 2017). Understanding how environmental factors influence the flowering transition of horticultural plants, as well as the underlying mechanisms involved, can help to improve the commercial value, lower production costs, and augment the annual production and seasonal supply of horticultural products (Higuchi 2018; Matsoukas et al. 2012).

Flowering time in the model plant *Arabidopsis* is regulated by integrating vernalization, temperature, photoperiod, hormones, age, autonomic pathways, and other floral transition signal transduction pathways (Srikanth and Schmid 2011; Cho et al. 2017). In 1936, M.K. Chailakhyan observed a type of flowering stimulator in *Chrysanthemum* that is produced in its leaves and transported to the shoot apical meristem (SAM) after photoperiod induction; it was designated ‘florigen’ (Chailakhyan and Krikorian 1975). This flowering element was later identified in *Arabidopsis* as a product of the *FLOWERING LOCUS T* (*FT*) gene (Kardailsky et al. 1999; Tsuji and Taoka 2014; Tsuji 2017). In *Arabidopsis*, TERMINAL FLOWER 1 (TFL1) of the FT/TFL1 family of proteins has been identified as a local floral inhibitor expressed in the SAM (Shannon and Meeks-Wagner 1991; Bradley et al. 1997). FT/TFL1 encodes a pair of flowering regulators that are homologous to phosphatidylethanolamine-binding proteins (PEBPs) (Ahn et al. 2006; Karlgren et al. 2011). The PEBP gene family in *Arabidopsis* includes six members: FT (Kim et al. 2013; Xu et al. 2012), TWIN SISTER OF FT (TSF) (Yamaguchi et al. 2005; Michaels et al. 2005; D'Aloia et al. 2011; Song et al. 2015), and MOTHER OF FT AND TFL1 (MFT) (Xi et al. 2010; Yoo et al. 2004) all promote flowering, whereas TFL1 (Kim et al. 2013), *Arabidopsis Thaliana* CENTRORADIALIS HOMOLOG (ATC) (Yoo et al. 2010; Huang et al. 2012) and BROTHER OF FT AND TFL1 (BFT) (Yoo et al. 2010) have function that differ from flowering.

**Fig. 1:**
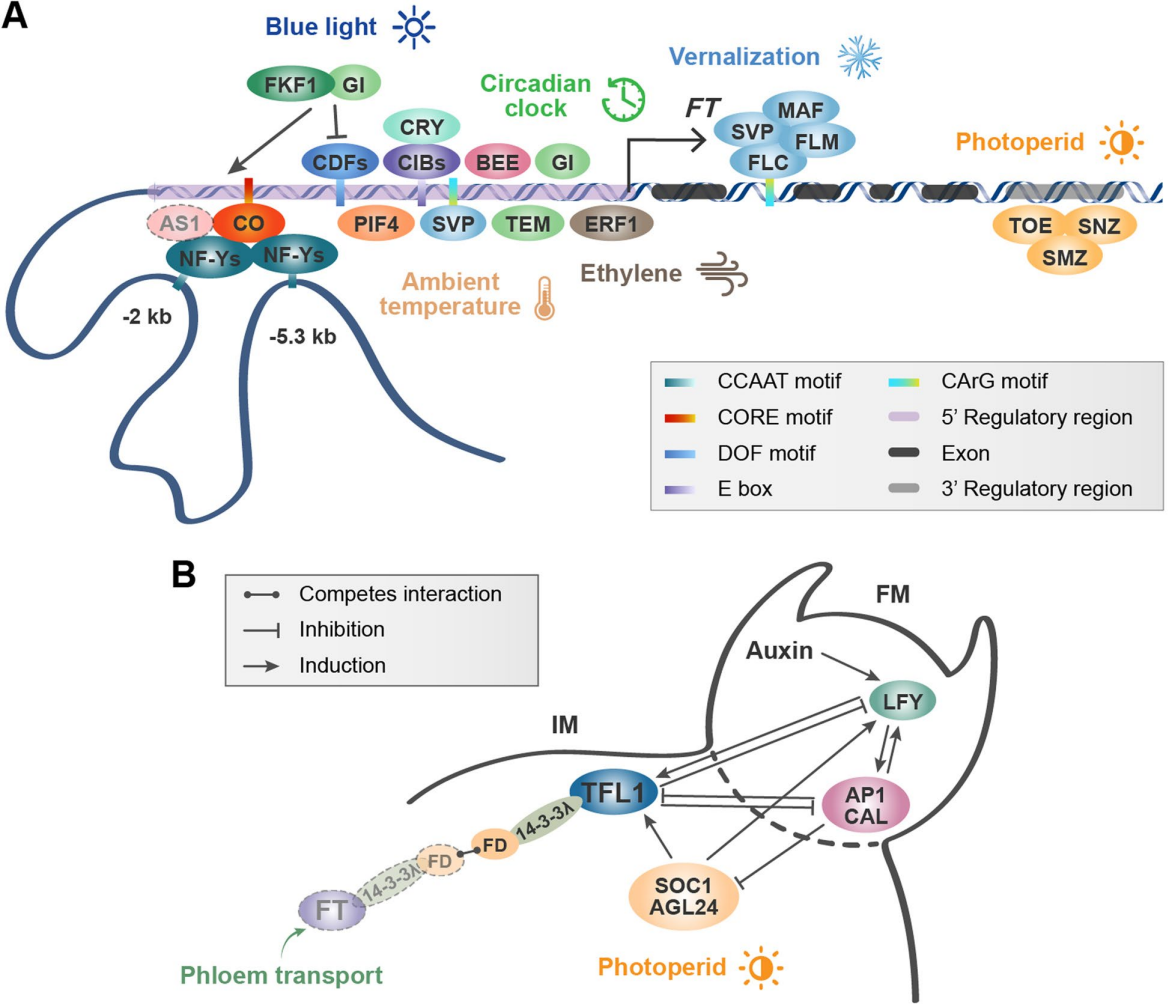
Transcriptional regulation of the *FT/TFL1* family genes by transcription factors in *Arabidopsis*

FT protein was induced in *Arabidopsis* leaf vascular tissue phloem companion cells and transferred to the SAM by interacting with FT-INTERACTING PROTEIN 1 (FTIP1), QUIRKY (QKY), and SYNTAXIN OF PLANTS121 (SYP121) (Mathieu et al. 2007; Liu et al. 2012; Putterill and Varkonyi-Gasic 2016; Liu et al. 2019). Long-distance transmission of the FT protein is blocked by its interaction with negatively-charged phosphatidylglycerol (PG) at low temperatures (Liu et al. 2020; Susila et al. 2021). After being transported to the SAM, the FT protein forms a complex with the bZIP transcription factor FD and induces the expression of the floral meristem-identity genes *APETALA1* (*AP1*) and *FRUITFULL* (*FUL*) (Abe et al. 2005; Wellmer and Riechmann 2010; Taoka et al. 2013). The interaction of environmental, endogenous, and hormonal signals precisely regulates the spatiotemporal expression of the *FT* gene in leaf phloem companion cells and the flowering in *Arabidopsis* (Fig. 1A). CONSTANS (CO) reflects the correspondence between external light signals and endogenous biological circadian clock, to activate the expression of *FT* at the right time to induce flowering (Imaizumi and Kay 2006; Song et al. 2015; Goralogia et al. 2017). Moreover, CYCLING DOF FACTORs (CDFs) directly bind to the proximal Block A region of the *FT* promoter to inhibit the transcription of *FT* (Imaizumi et al. 2005; Goralogia et al. 2017). Genes related to the circadian clock, temperature, and blue-light signals, such as GIGANTEA (GI) (Sawa and Kay 2011), BR ENHANCED Production 1 (BEE1) (Wang et al. 2019), PHYTOCHROME INTERACTING FACTOR 4 (PIF4) (Kumar et al. 2012), and CIB (cryptochrome-interacting basic-helix-loop-helix) (Liu et al. 2008), bind upstream from the transcription start site (TSS) of the *FT* gene, triggering its expression. TEMPRANILLO (TEM) (Castillejo and Pelaz 2008), TARGET OF EAT 1 (TOE1), TOE2, SCHAFLMüTZE (SMZ), SCHNARCHZAPFEN (SNZ) (Mathieu et al. 2009), and SHORT VEGETATIVE PHASE (SVP) (Lee et al. 2007) respond to ambient temperature or photoperiod to directly repress *FT* expression. Further, several MADS transcription factors, namely FLOWERING LOCUS C (FLC), SVP, FLOWERING LOCUS M (FLM), and MADS AFFECTING FLOWERING (MAF), can inhibit transcription by binding to the first intron of *FT* at low temperatures or before vernalization (Luo et al. 2021). Other hormone signals also play a role in controlling the initiation of flowering. For example, ERF1, a key member of the ethylene signal transduction pathway, binds directly to the *FT*’s promoter and inhibits its transcription (Chen et al. 2021). Furthermore, polycomb group (Pc-G) proteins reportedly mediate epigenetic gene regulation, which maintains the identity of the inflorescence and floral meristems after floral induction (Müller-Xing et al. 2014). The simultaneous occurrence of H3K27me3 at *FT* has also been demonstrated, using a sequential ChIP analysis (Jiang et al. 2008). The genes of PcG subunits, including EMBRYONIC FLOWER 2 (EMF2), EMF1, CURLY LEAF (CLF), MULTICOPY SUPPRESSOR OF IRA 1 (MSI1) and LIKE HETEROCHROMATIN PROTEIN 1 (LHP1), deposit H3K27me3 in the chromatin of *FT* to repress its expression (Jiang et al. 2008; Schatlowski et al. 2008; Mozgova and Hennig 2015; Merini and Calonje 2015). Unlike other PcG target genes in *Arabidopsis*, modification by H3K27me3 occurs in the promoter, coding region, and downstream region of the *FT* gene (Turck et al. 2007).

In *Arabidopsis*, the antagonism of *TFL1* and floral meristem-specific genes controls flower initiation and the ensuing inflorescence structure. *TFL1*, which is expressed in the central region of the apical meristem but can move to the meristem layer L1, is required for movement into the SAM to regulate floral transition (Conti and Bradley 2007; Goretti et al. 2020). Once in the SAM, TFL1 interacts with the bZIP transcription factor FD via the 14–3-3 protein, and FT and TFL1 compete for binding FD to regulate the downstream floral meristem identification genes *LEAFY* (*LFY*), *APETALA1* (*AP1*), and *CAULIFLOWER* (*CAL*), which maintains meristem indeterminacy (Zhu et al. 2020; Goretti et al. 2020) (Fig. 1B). Under long day (LD) conditions, SOC1 and AGL24 bind to *TFL1*’s chromatin regions and directly activate its transcription in the SAM (Azpeitia et al. 2021). LFY binds to the *TFL1* promoter and directly activates *TFL1* transcription, a regulatory loop which ensures that flower formation occurs only when *AP1/CAL* levels are sufficiently high to repress *TFL1* expression and trigger the genetic program required for flower development (Serrano-Mislata et al. 2017). The proteins encoded by the *FT/TFL1* family of genes have small differences in conformation, giving them opposite functions in plants. Hanzawa et al. (2005) showed that changing only a single amino acid in the *Arabidopsis* TFL1 protein can render TFL1 to function as a floral activator, and vice versa.

Recent reports in gymnosperms indicate that the ancestor of *FT* functioned in a *TFL1-*like manner (Karlgren et al. 2011). For example, *PaFTL1* and *PaFTL2* in Norway spruce (*Picea abies*) both repress this conifer tree’s growth, and their heterologous expression in *Arabidopsis* also delays the onset of flowering (Karlgren et al. 2011; Klintenäs et al. 2012). The data to date suggests that the function of *FT* and *TFL1* diverged after the evolutionary separation in different plant species. Moreover, *FT/TFL1* family genes are reportedly involved in other developmental processes and feature functional diversification in regulating flowering time in several species (Karlgren et al. 2011; Klintenäs et al. 2012). This review summarizes the functional diversification and molecular mechanisms of the *FT/TFL1* family members in horticultural plants, to provide a timely reference for further research on these pivotal genes in horticultural plants.

### *FT/TFL1* family genes regulate flowering time in horticultural plants

In horticultural plants, the number of *FT/TFL1* family genes varies yet they have conserved functions in the process of floral transition (Table 1). For example, in ornamental plants of the Asteraceae—the largest flowering plant family contains the greatest number of species—three *FT-like* genes have been identified in *Chrysanthemum seticuspe*: *CsFTL1*, *CsFTL2*, and *CsFTL3*, which are flowering inducers (Oda et al. 2012; Mao et al. 2016; Sun et al. 2017; Wang et al. 2020a). Mao et al. (2016) found that the archetypal and alternative splicing (AS) forms of *CmFTL1* (*C. morifolium* cultivar ‘Jimba’) has the function of complementing the late-flowering phenotype of the *Arabidopsis ft-10* mutant, and *CmFTL1* can induce flowering in *C. morifolium* ‘Yuuka’ (Wang et al. 2020a). Sucrose induces the transcriptional upregulation of *CmFTL2* (*C. morifolium* ‘Yuuka’) in chrysanthemum leaves and promotes flowering (Sun et al. 2017). However, changing a single amino acid in *CmFTL3* of chrysanthemum results in the loss of its flowering function (Sun et al. 2018). Moreover, there are three *TFL1* homologs in chrysanthemum that act as flowering inhibitors (Higuchi and Hisamatsu 2015; Gao et al. 2019; Haider et al. 2020). The orchid family (Orchidaceae) is the second largest family of flowering plants, for which three *FT-like* genes were identified in *Phalaenopsis*: *PhFT1*, *PhFT3*, and *PhFT5,* heterologous expression of which in *Arabidopsis* cause early flowering phenotype (Zhou et al. 2018; Li et al. 2014). Ectopic expression of *OnFT* could not fully complement the late-flowering phenotype of the *Arabidopsis ft-1* mutant, and it was not regulated by photoperiod but did inhibit flowering in *Oncidium* (Hou and Yang 2009). Six PEBP family genes (i.e., *DhFT3*, *DhFT1*, *DhMFT*, *DhTFL1b*, *DhFT2*, and *DhTFL1a*) were isolated and characterized from the *Dendrobium huoshanense* genome (Song et al. 2021). Gibberellin (GA) treatment increased the expression of all those *DhFTs* and promoted flowering, while inhibiting the expression of *DhTFL1s* (Song et al. 2021). A year-round tropical orchid, *Arundina graminifolia*, lacks any *TFL1-like* gene, but does have two *FT-like* genes: *AgFT1* and *AgFT2.* The functions of *AgFT1* and *AgFT2* in regulating flowering have not been verified, however (Auberon et al. 2016; Ahmad et al. 2021).

**Table 1:** Diverse functions of the *FT/TFL1* gene family in horticultural plants

Gene name	Effect on flowering	Other function	Regulatory input	References
Arabidopsis (***Arabidopsis thaliana***)				
AtFT	Induction	Plant growth; Dormancy	LD	Shannon and Meeks-Wagner, 1991; Yoo et al., 2004; Yamaguchi et al., 2005; Michaels et al., 2005; Xi et al., 2010; Yoo et al., 2010; D'Aloia et al., 2011; Huang et al., 2012; Kim et al., 2013; Moraes et al., 2019; Azpeitia et al., 2021; Chen et al., 2021
AtTSF	Induction		LD	
AtTFL1	Inhibition	Inflorescence development	LD	
AtBFT	Inhibition	Inflorescence development	LD	
AtATC	Inhibition			
AtMFT	Induction	Seed germination		
Chrysanthemum (***Chrysanthemum*** spp.)				
CsFTL1	Induction		LD, NB	Oda et al., 2012; Higuchi et al., 2013; Higuchi and Hisamatsu, 2015; Mao et al., 2016; Sun et al., 2017; Gao et al., 2019; Wang et al., 2020a, b; Haider et al., 2020
CsFTL2	Induction		LD, Sucrose	
CsFTL3	Induction		LD, Sucrose	
CsTFL1b	Inhibition			
CmTFL1a	Inhibition			
CmTFL1c	Inhibition			
CsAFT	Inhibition		LD, NB	
Phalaenopsis (***Phalaenopsis****hybrid*)				
PhFT1	Induction			Li et al., 2014; Zhou et al., 2018; Jiang et al., 2022
PhFT3	Induction			
PhFT5	Induction			
PhFT6	Inhibition			
Orchid (***Oncidium****Gower Ramsey*)				
OnFT	Induction			Hou and Yang, 2009
OnTFL1	Inhibition			
Dendrobium (***Dendrobium huoshanense***)				
DhFT3	Induction		Gibberellin	Song et al., 2021
DhFT1	Induction		Gibberellin	
DhFT2	Induction		Gibberellin	
DhTFL1a	Inhibition			
DhTFL1b	Inhibition			
Tomato (***Solanum lycopersicum***)				
SP	Inhibition	Indeterminate growth		Molinero-Rosales et al., 2004; Lifschitz et al., 2006; Shalit et al., 2009; Jiang et al., 2013; Lifschitz et al., 2014; Cao et al., 2016; Song, 2020
SFT	Induction	Inflorescence development		
FTL1	Induction		SD	
SP5G	Inhibition			
SP5G2	Inhibition			
SP5G3	Inhibition			
Strawberry (***Fragaria****×****ananassa***)				
FvFT1	Induction		LD	Koskela et al., 2012; Nakano et al., 2015
FvTFL1	Inhibition		LD, Cool temperature	
Rose (***Rosa*** spp.)				
RoKSN	Inhibition			Iwata et al., 2012; Randoux et al., 2014, b; Otagaki et al., 2015
RoFT	Induction			
Poplar (***Populus*** spp.)				
PtFT1	Induction			Mohamed et al., 2010; Hsu et al., 2011; Gómez-Soto et al., 2022
PtFT2	Inhibition	Vegetative growth; Shoot dormancy	LD, High temperature	
PopCEN1	Inhibition			
PopCEN2	Inhibition			
Sugar beet (*Beta vulgaris*)				
BvFT1	Inhibition		SD, Non-vernalized	Pin et al., 2010
BvFT2	Induction			
Cucumber (*Cucumis sativus*)				
‘short-1’ UR CsFT	Induction			Wen et al., 2019; Wang et al., 2020b
‘short-2’ UR CsFT	Induction			
‘long’ UR CsFT	Inhibition			
CsTFL1	Inhibition	Determinate growth		
Tulip (*Tulipa gesneriana*)				
TgFT1	Inhibition			Leeggangers et al., 2018
TgFT2	Induction			
TgFT3	Inhibition			
Potato (*Solanum tuberosum*)				
StSP5G	Inhibition			Navarro et al., 2011; Lee et al., 2013
StSP5G-like	Inhibition			
StSP6A		Tuber formation		
Onion (*Allium cepa*)				
AcFT1		Bulb formation	LD	Blackman et al., 2010; Lee et al., 2013
AcFT2	Induction		Vernalization	
AcFT4	Inhibition	Bulb formation		
Kiwifruit (*Actinidia* spp.)				
Kiwifruit FT	Induction	Dormancy release	Cool temperature	Varkonyi-Gasic et al., 2013
Kiwifruit CEN	Inhibition			
Pineapple (*Ananas comosus*)				
AcFTL2	Induction		Ethylene	Liu and Fan, 2016; Liu et al., 2018

In vegetable crops, the FT-like protein StSP3D is essential for flowering in potato (*Solanum tuberosum*) (Navarro et al. 2011). Tomato (*Lycopersicon esculentum*) is the second most globally important vegetable crop (after potato), whose flowering time is jointly controlled by the flowering inducer *SINGLE FLOWER TRUSS* (*SFT*) and suppressor *SELF PRUNING* (*SP*) (Molinero-Rosales et al. 2004; Jiang et al. 2013). *SFT* is an ortholog of *FT-like* that is expressed in mature leaves and systematically promotes flowering, while *SP* is the ortholog of *TFL1-like* that is instead expressed in young leaves and shoot tips, and inhibits flowering (Shalit et al. 2009). Recently, *FTL1*, which regulates flowering time in tomato, was located and sequenced through map-based cloning. *FTL1* is only specifically expressed under short day (SD) conditions and regulates tomato flowering by promoting the expression of *SFT* (Song 2020). Among fruit crops, in strawberry (*Fragaria* × *ananassa*) *FvFT1* and *FvTFL1* have antagonistic functions for inducing flowering. Interestingly, owing to a base deletion in *FvTFL1*, strawberry has since become a permanent flowering plant (Koskela et al. 2012; Nakano et al. 2015).

Moreover, perennial woody plants possess flowering-inductive *FT* genes and flowering-inhibitory *TFL1* genes. Poplar (*Populus species*) contains two *FT-like* genes (*PtFT1/PtFT2*) (Hsu et al. 2011) and two *CEN/TFL1-like* genes (*PopCEN1/PopCEN2*) (Mohamed et al. 2010). Overexpression of *PtFT1* in *Poplar* caused its flowering in the tissue culture stage at 6 weeks; hence, *PtFT1* promotes flowering (Hsu et al. 2006; Hsu et al. 2011; Gómez-Soto et al. 2022). Both *PopCEN1* and *PopCEN2* inhibited flowering, however. Downregulating the expression of *PopCEN1* and *PopCEN2* can accelerate the time of first onset of flowering and the maturity of poplar (Mohamed et al. 2010). In a Chinese continuous-flowering rose plant cultivar, the *TFL1-like* gene *RoKSN* is a flowering suppressor whereas *RoFT* is a floral inducer (Iwata et al. 2012; Otagaki et al. 2015). The insertion of a retrotransposon in *RoKSN* inhibits *RoKSN* expression in roses, thereby facilitating their continuous flowering (Randoux et al., 2014, b). Collectively, these reports suggest the function of *FT/TFL1* family genes is generally conserved in horticultural plants.

### Functional diversification of FT/TFL1-like in horticultural plants

The functions of proteins encoded by homologous *FT/TFL1-like* genes are not entirely conserved in horticultural plants, in that they show functional diversification in regulating flowering time (Table 1).

Homologous genes of *FT* could contribute to inhibiting flowering. In vegetable crops, the two *FT-like* homologous genes *BvFT1* and *BvFT2* in sugar beet (*Beta vulgaris*) function antagonistically in flowering. Under non-vernalized or SD conditions, the flowering inhibitor *BvFT1* inhibits flowering by limiting the expression of the flowering-inducing factor *BvFT2* (Pin et al., 2010). In cucumber (*Cucumis sativus*), the structural types in the upstream region (UR) of *CsFT* have differential effects on flowering induction; the ‘short-1’ UR *CsFT* and ‘short-2’ UR *CsFT* accelerate the onset of flowering, whereas the ‘long’ UR *CsFT* delays flowering (Wang et al., 2020b). Four *FT-like* homologous genes have been identified in tomato: *SP3D/SFT* has a florigen function, whereas *SP5G*, *SP5G2*, and *SP5G3* are characterized by flowering inhibitory activity (Cao et al., 2016). *AcFT4* in onion (*Allium cepa*) and both *StSP5G* and *StSP5G-like* in potato are also able to inhibit flowering (Navarro et al., 2011; Lee et al., 2013). In ornamental plants, three PEBP genes were isolated in tulip (*Tulipa gesneriana*): *TgFT1*, *TgFT2*, and *TgFT3*. Overexpression of *TgFT2* in *Arabidopsis* resulted in an early-flowering phenotype, while *TgFT1* and *TgFT3* overexpression resulted in a late-flowering phenotype (Leeggangers et al., 2018). The *PhFT6* in *Phalaenopsis* and *HaFT1* in sunflower (*Helianthus annuus*) can also repress their flowering (Li et al., 2014; Blackman et al., 2010). The two *FT-like* genes *PtFT1/PtFT2* in poplar, a woody perennial species, also have opposing flowering regulatory functions (Hsu et al., 2011; Mohamed et al., 2010). *PtFT1* has a florigen function, whereas *PtFT2*, it induced by LDs and high temperature, reduces the level of GA via the GA 13-hydroxylation pathway and maintains the vegetative growth of poplar to preclude flowering (Gómez-Soto et al., 2022).

In addition to regulating flowering time, members of *FT/TFL1-like* genes are involved in a variety of other processes in horticultural plants. *CsTFL1* inhibits determinate growth and terminal flower formation in cucumbers (Zhao et al., 2018; Wen et al., 2019; Njogu et al., 2020). Navarro et al. (2011) reported that overexpressing the *FT* homologous gene *Hd3a* in potato enabled it to grow more tubers than the wild type, and that the endogenous gene *StSP6A* also had a similar function, thus indicating that *FT* promotes tuber formation. In onion, LDs induced the downregulation of *AcFT4* expression but the upregulation of *AcFT1* expression, which promoted the formation of bulbs and increased the yield (Lee et al., 2013). In tomato *sft* mutants, the inflorescence differentiated into only one flower, the sepals were enlarged, and leaves have excess intercalary leaflets; however, the leaves became smaller blades and lack folioles after the overexpression of *SFT* (Shalit et al., 2009; Lifschitz et al., 2014). In *Dendrobium* Chao Praya Smile, *DoFT*-RNAi transgenic lines also displayed abnormal inflorescence development and delayed pseudobulb formation, suggesting that *DOFT* may have evolved with unknown functions related to the regulation of storage organs and flower development (Wang et al., 2017). *PtFT2* promotes vegetative growth and shoot dormancy in poplar trees (Mohamed et al., 2010); similarly, *FT* and *CEN* are involved in the regulation of kiwifruit plant growth by integrating developmental and environmental signals (Varkonyi-Gasic et al., 2013). Taken together, these reports show that the functions of members of the *FT/TFL1* gene family have evolved dynamically over the course of horticultural plants’ evolution.

### Regulation of *FT/TFL1* family genes in horticultural plants

**Fig. 2:**
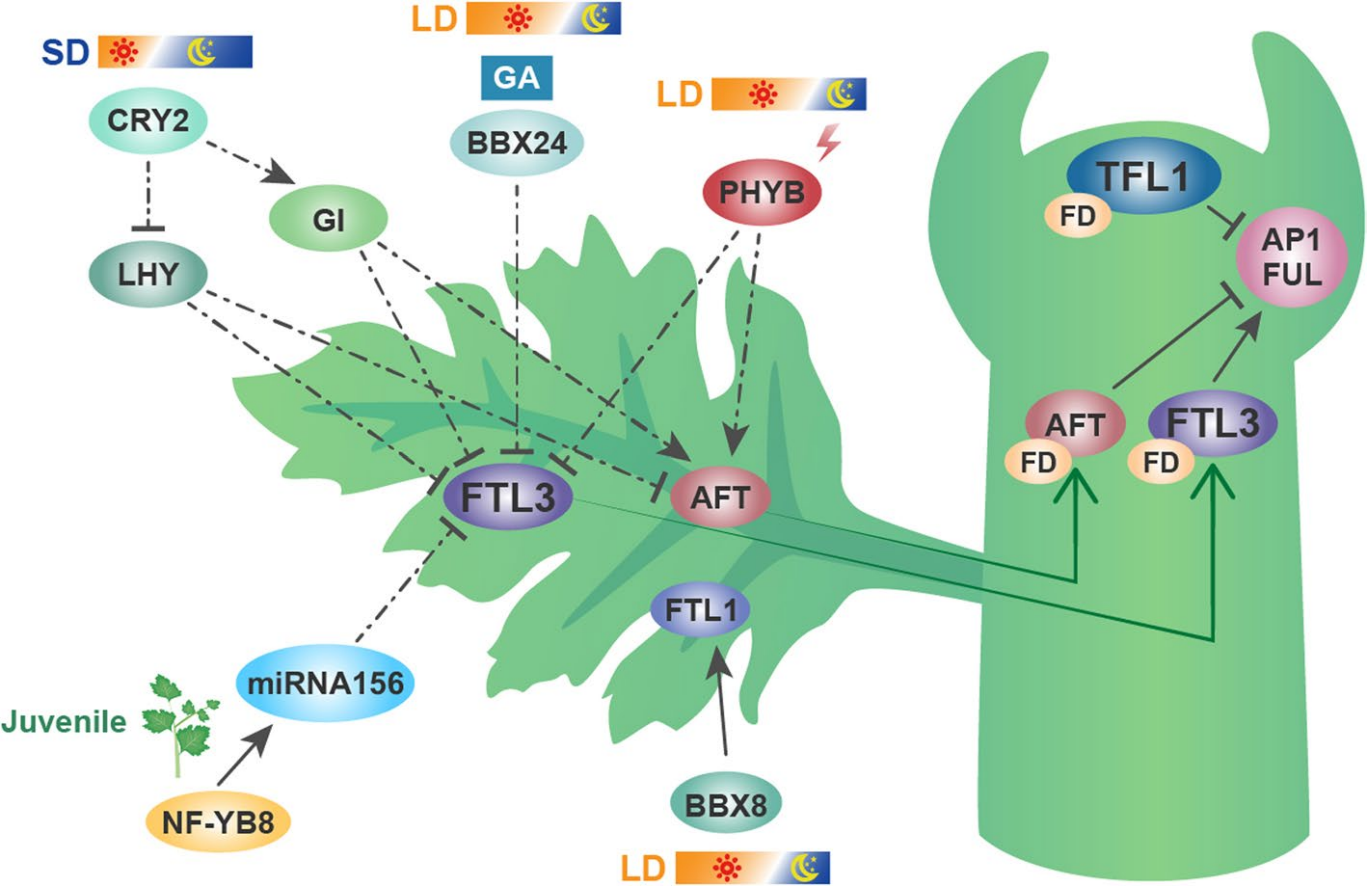
Flowering time regulation by florigen and anti-florigen in *Chrysanthemum*

The photoperiodic pathway is the most important and most conserved of the floral induction pathways, and some of the key loci and mechanisms are shared even among distantly related plant species, whereas others are not conserved and give rise to crucial species differences (Matsoukas et al., 2012). The autumn flowering chrysanthemum cultivars are short day plants that require a repeated SD photoperiod for successful flowering, because *CsFTL3* expression increases with such repeated SDs before successful flowering occurs, but their vegetative growth can be strictly maintained under LD or night-break (NB) conditions. When SDs switch to LDs before the involucre-forming stage, those plants do not initiate florets on the apical receptacle, or their capitulum development is strongly suppressed (Higuchi, 2018; Nakano et al., 2019). Recent studies have revealed the transcriptional regulation mechanism of the *FT/TFL1* family genes in chrysanthemum (Fig. 2). *ClCRY2* facilitates floral transition in *C. lavandulifolium* by fine-tuning the expression of circadian clock-related genes, such as the downregulation of *LHY* and overexpression of *GI* (Yang et al., 2018). By downregulating *CsFTL3* and *CsAFT*, CsLHY-SRDX induced a photoperiod-insensitive floral transition (Oda et al., 2017). The transcription of another circadian-clock-related gene, *CsGI*, has been shown to increase the necessary night length for blooming, chiefly by maintaining lower levels of *CsAFT* (Oda et al., 2020). In chrysanthemums, *CsPHYB*-mediated light signaling upregulates *CsFTL3* but downregulates *CsAFT* to determine their obligate photoperiodic blooming response (Higuchi et al., 2013). Furthermore, gibberellins function critically in floral induction in response to LDs (Porri et al., 2012). *CmBBX24* inhibits the expression of *CmFTL3*, which regulates flowering primarily through effects on the GA pathway under LDs (Yang et al., 2014). Recently, the role of NF-Y proteins in the aging pathway in chrysanthemum was identified, in that *CmNF-YB8* influences flowering time by directly upregulating the expression of *cmo*-*MIR156* in the aging pathway (Wei et al., 2017). More recently, the CO homologous protein CmBBX8 was discovered to target *CmFTL1* for flowering regulation in chrysanthemum (Wang et al., 2020a).

**Fig. 3:**
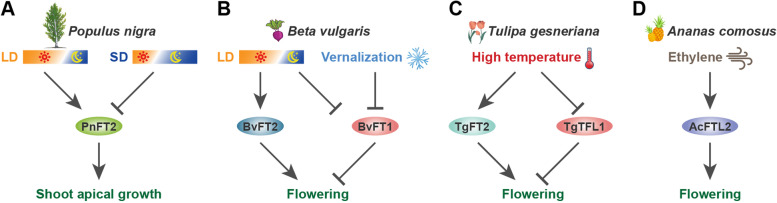
Flowering time regulation by florigen and anti-florigen in *Populus nigra* (**A**), *Beta vulgaris* (**B**), *Tulipa gesneriana* (**C**), and *Ananas comosus* (**D**). Arrows depict induction and dashed lines indicate inhibition

Nevertheless, the functions of *CO1* and *CO2* in poplar do not overlap with those in *Arabidopsis*. The growth of *CO2* RNAi transgenic poplar stopped when LDs transitioned to SDs, and its shoots formed earlier. Overexpression of *CO1* and *CO2* in poplar did not induce the upregulated expression of *FT2* under SD conditions, and its timing of flowering and bud formation did not change (Hsu et al., 2012). Another SD-dependent *FT2* inhibition pathway mediated by *LHY2* was recently discovered in poplar. Under SD conditions, *LHY2* is induced to express and directly bind to the homeopathic element at the 3′ end of *FT2* to inhibit its expression, resulting in the arrested growth of poplar (Fig. 3A). But under LD conditions, the expression of *LHY2* is low, while CO and other activators induce the expression of *FT2*, thereby promoting the flowering of poplar (Ramos-Sánchez et al., 2019; André et al., 2022).

Some *FT* homologs regulate flowering and aspects of development in response to temperature except for photoperiod in horticultural plants. In sugar beet, the expression of the flowering inhibitor *BvFT1* was inhibited by both vernalization and LD conditions and *BvFT2* was induced under LDs (Pin et al., 2010) (Fig. 3B). In strawberry, *FvFT1* was specifically upregulated in mature leaves and this promoted the upregulation of *FvSOC1* in shoot tips, which activated *FvTFL1* expression to inhibit flowering under LDs (Mouhu et al., 2013; Rantanen et al., 2014). Furthermore, *FvTFL1* was regulated by a temperature-dependent pathway independent of photoperiod-dependent regulation (Rantanen et al., 2015). Floral transition of lily (*Lilium longiflorum*) is also induced by low-temperature conditions and is not regulated by photoperiod. In lily, *LlFT*, as a flowering activator, is significantly induced by a period of low-temperature treatment and this promoted flowering; however, without the vernalization treatment, overexpression of *LlFT* also led to a bloom, indicating that *LlFT* is the main regulatory factor controlling flowering in the vernalization pathway (Lazare and Zaccai et al., 2016). After switching from vegetative to reproductive growth, *LlFT* expression was further reduced in floral meristems and small flower buds. Therefore, *LlFT* is thought to be involved in switching the meristem to a flowering state during vernalization, but it does not act as a flowering inducer (Leeggangers et al., 2018). Further, the flowering regulation of *Narcissus tazetta* and tulip is induced by high temperature and does not depend on the photoperiod and vernalization pathways. In narcissus, high temperature induces the transcription of *Narcissus FLOWERING LOCUST*1 (*NFT1*), which promotes the expression of the downstream *LFY* homolog gene *NLF* and induces flowering (Li et al., 2013; Noy-Porat et al., 2013). The floral transition of tulip occurs in the bulb, for which high temperature induces the expression of *TgFT2* and inhibits the expression of *TgTFL1*, which then induces the floral transition (Leeggangers et al., 2018) (Fig. 3C). On the contrary, the chrysanthemum flowering is severely delayed by high temperature during the summer, when the reduction of *CsFTL3* expression at high temperatures are involved in flowering’s retardation in *C. seticuspe* (Higuchi, 2018). Interestingly, exogenous ethylene induces the upregulated expression of *FT-like* and *AP1-like* genes, which promotes transition to flower formation in pineapple (Liu and Fan, 2016; Liu et al., 2018) (Fig. 3D), this starkly differing from their inhibitory effect on *Arabidopsis* flowering (Chen et al., 2021). These findings indicate that not only the functions of members of the *FT/TFL1* family of genes, but also their upstream regulators, have evolved drastically and in cases also divergently in horticultural plants.

The transport of florigens in horticultural plants has also been studied. In *Cucurbita moschata*, LD-induced transport of FT proteins from its leaves to shoot tips promoted the transition into flowering (Lin et al., 2007). Moreover, floral promotion via the graft transmission of FT has been demonstrated in woody plants. For a recent example, when the scion of *JcFT*-RNAi transgenic *Jatropha curcas* was grafted onto *SUC2:JcFT* rootstock, FT protein was transported into the scion which promoted the transition into flowering, whose efficacy depended on the length of the scion (Tang et al., 2022). In trifoliate orange, its early flowering was induced in the transgenic tomato as well as trifoliate orange plants transformed with *ToFT*. However, the rootstocks of transgenic trifoliate orange could not induce flowering of grafted wild-type (WT) juvenile scions because of their low accumulation of total FT protein (Wu et al., 2022). That finings suggests the expression of *FT* must reach a certain threshold to induce flowering. A *TFL1* homolog (*RoKSN*) in rose was found to be immobile, precluding its transmission via grafting experiments (Randoux et al., 2014, b). Yet when a WT chrysanthemum plant was grafted onto the *CsTFL1*-ox stock, the flowering of the WT scion was delayed vis-à-vis the WT/WT grafting (Higuchi and Hisamatsu, 2015). Spatial expression patterns of *CsTFL1* showed that it was mainly expressed in shoot tips, with low expression levels in leaves (Higuchi and Hisamatsu, 2015; Gao et al., 2019; Haider et al., 2020). These results suggest that *CsTFL1* probably can move long distances through a grafting union as a floral repressor, to systemically regulate an indeterminate apical meristem. Because FT participates in vesicle trafficking (Liu et al., 2020), whether the transport of TFL1 occurs via a similar way awaits investigation.

#### Outlook

In summary, many studies of diverse horticultural plants have revealed the conserved functioning of members of the *FT/TFL1* gene family, which have evolved dynamically over the course of horticultural plant evolution. Moreover, amino acid substitutions in *FT/TFL1* family genes in *Arabidopsis* and horticultural plants such as sugar beet could cause a conversion in functionality, from having repressor activity to becoming a floral activator and vice versa (Ho and Weigel, 2014; Pin et al., 2010). A single base deletion or the products of a premature stop codon in *TFL1* gene in strawberry facilitates their continuous flowering (Koskela et al., 2012). In cucumber and domesticated tomato, the short upstream region of *CsFT* and mutations in the *cis*-regulatory region of antiflorigen *SP5G* hasten their onset of flowering, respectively (Soyk et al., 2017; Wang et al., 2020b). These results suggest *FT/TFL1* family genes are elite editing targets for manipulating gene structure, to change key flowering characteristics of horticultural plants, using genome editing technology, which is a powerful and precision-breeding approach, although there are legal/ethical concerns (Gao, 2021).

Because *FT/TFL1* family genes integrate multiple regulatory pathways, such as photoperiod, vernalization, and ambient temperature pathway, to govern flowering, not only the functions of their members but also their upstream regulators have drastically evolved in horticultural plants. With ongoing global warming, the rise in ambient temperature is often accompanied by a greater concentration of carbon dioxide (CO_2_), which is conducive to the accumulation of photosynthetic products (sugar and starch) in plants. This increase in CO_2_ is apt to cause changes in tissues’ sugar status or directly drives *FT/TFL1* to regulate flowering (Jagadish et al., 2016). Although studies have found that *FT* can mediate nitrogen’s control of flowering, its regulatory mechanism is still not well understood (Gras et al., 2018; Zhang et al., 2021). Therefore, elucidating in detail how the regulation mechanisms of *FT/TFL1* family genes may respond to various environmental and endogenous stimuli would promote the development of an efficient and energy-saving approach to regulate flowering. Due to the rapid adoption and spread of genomic sequencing technology applied to horticultural plants, genomic resources are becoming increasingly available. This combined with other techniques, namely high-throughput phenotyping, genomic selection, and gene function analysis, will enable us to obtain detailed knowledge of the *FT/TFL1* gene family, so as to modify their action to meet the increasing demand for horticultural products in the future.

## Data Availability

Not applicable.

## Abbreviations

SAM: Shoot apical meristem
*FT*: *FLOWERING LOCUS T*
*AP1*: *APETALA1*
*FUL*: *FRUITFULL*
SD: Short-day
DN: Day-neutral
TSS: Transcription start site
*TFL1*: *TERMINAL FLOWER 1*
LD: Long*-*day
PEBPs: Phosphatidylethanolamine-binding proteins
*MFT*: *MOTHER OF FT AND TFL1*
*TSF*: *TWIN SISTER OF FT*
*ATC*: *Arabidopsis thaliana CENTRORADIALIS homolog*
*BFT*: *BROTHER OF FT AND TFL1*
NB: Night-break
GA: Gibberellin
*SFT*: *SINGLE FLOWER TRUSS*
*SP*: *SELF PRUNING*
UR: Upstream region
*NF-Y*: *Nuclear Factor Y*
*AS1*: *ASYMMETRICLEAVES 1*
*TEM*: *TEMPRANILLO*
*TOE1*: *TARGET OF EAT 1*
*SMZ*: *SCHAFLMüTZE*
*SNZ*: *SCHNARCHZAPFEN*
*BEE1*: *BR ENHANCED EXPRESSION 1*
*SVP*: *SHORT VEGETATIVE PHASE*
*PIF4*: *PHYTOCHROME INTERACTING FACTOR 4*
*FLC*: *FLOWERING LOCUS C*
*FLM*: *FLOWERING LOCUS M*
*MAF*: *MADS AFFECTING FLOWERING*
IM: inflorescence meristem
*LFY*: *LEAFY*
*CAL*: *CAULIFLOWER*
FM: floral meristem
*SOC1*: *SUPPRESSOR OF OVEREXPRESSION OF CO 1*
*AGL24*: *AGAMOUS LIKE 24*
*NFT1*: *FLOWERING LOCUST1*
FTIP1: FT-INTERACTING PROTEIN 1
*NaKP1*: *SODIUM POTASSIUM ROOT DEFECTIVE 1*
PG: Phosphatidylglycerol
PcG: Polycomb group
*LHP1*: *LIKE HETEROCHROMATIN PROTEIN 1*
*CLF*: *CURLY LEAF*
*EMF2*: *EMBRYONIC FLOWER 2*
*MSI1*: *MULTICOPY SUPPRESSOR OF IRA 1*

## References

[CR1] Abe M, Kobayashi Y, Yamamoto S, Daimon Y, Yamaguchi A, Ikeda Y (2005). FD, a bZIP protein mediating signals from the floral pathway integrator FT at the shoot apex. Science..

[CR2] Ahmad S, Lu C, Gao J, Ren R, Wei Y, Wu J (2021). Genetic insights into the regulatory pathways for continuous flowering in a unique orchid Arundina graminifolia. BMC Plant Biol.

[CR3] Ahn JH, Miller D, Winter VJ, Banfield MJ, Lee JH, Yoo SY (2006). A divergent external loop confers antagonistic activity on floral regulators FT and TFL1. EMBO J.

[CR4] André D, Zambrano JA, Zhang B, Lee KC, Rühl M, Marcon A (2022). Populus SVL acts in leaves to modulate the timing of growth cessation and bud set. Front Plant Sci.

[CR5] Auberon F, Olatunji OJ, Krisa S, Antheaume C, Herbette G, Bonté F (2016). Two new Stilbenoids from the aerial parts of Arundina graminifolia (Orchidaceae). Molecules..

[CR6] Azpeitia E, Tichtinsky G, Le Masson M, Serrano-Mislata A, Lucas J, Gregis V (2021). Cauliflower fractal forms arise from perturbations of floral gene networks. Science..

[CR7] Blackman BK, Strasburg JL, Raduski AR, Michaels SD, Rieseberg LH (2010). The role of recently derived FT paralogs in sunflower domestication. Curr Biol.

[CR8] Bradley D, Ratcliffe O, Vincent C, Carpenter R, Coen E (1997). Inflorescence commitment and architecture in Arabidopsis. Science..

[CR9] Cao K, Cui L, Zhou X, Ye L, Zou Z, Deng S (2016). Four tomato FLOWERING LOCUS T-like proteins act antagonistically to regulate floral initiation. Front Plant Sci.

[CR10] Castillejo C, Pelaz S (2008). The balance between CONSTANS and TEMPRANILLO activities determines FT expression to trigger flowering. Curr Biol.

[CR11] Chailakhyan MK, Krikorian A (1975). Forty years of research on the hormonal basis of plant development—some personal reflections. Bot Rev.

[CR12] Chen Y, Zhang L, Zhang H, Chen L, Yu D (2021). ERF1 delays flowering through direct inhibition of FLOWERING LOCUS T expression in Arabidopsis. J Integr Plant Biol.

[CR13] Cho LH, Yoon J, An G (2017). The control of flowering time by environmental factors. Plant J.

[CR14] Conti L, Bradley D (2007). TERMINAL FLOWER1 is a mobile signal controlling Arabidopsis architecture. Plant Cell.

[CR15] D'Aloia M, Bonhomme D, Bouché F, Tamseddak K, Ormenese S, Torti S (2011). Cytokinin promotes flowering of Arabidopsis via transcriptional activation of the FT paralogue TSF. Plant J.

[CR16] Gao C (2021). Genome engineering for crop improvement and future agriculture. Cell..

[CR17] Gao Y, Gao Y, Wu Z, Bu X, Fan M, Zhang Q (2019). Characterization of TEMINAL FLOWER1 homologs CmTFL1c gene from Chrysanthemum morifolium. Plant Mol Biol.

[CR18] Gómez-Soto D, Allona I, Perales M (2022). FLOWERING LOCUS T2 promotes shoot apex development and restricts internode elongation via the 13-hydroxylation gibberellin biosynthesis pathway in poplar. Front Plant Sci.

[CR19] Goralogia GS, Liu TK, Zhao L, Panipinto PM, Groover ED, Bains YS (2017). CYCLING DOF FACTOR 1 represses transcription through the TOPLESS co-repressor to control photoperiodic flowering in Arabidopsis. Plant J..

[CR20] Goretti D, Silvestre M, Collani S, Langenecker T, Méndez C, Madueño F (2020). TERMINAL FLOWER1 functions as a Mobile transcriptional cofactor in the shoot apical meristem. Plant Physiol.

[CR21] Gras DE, Vidal EA, Undurraga SF, Riveras E, Moreno S, Dominguez-Figueroa J (2018). SMZ/SNZ and gibberellin signaling are required for nitrate-elicited delay of flowering time in Arabidopsis thaliana. J Exp Bot.

[CR22] Haider S, Gao Y, Gao Y (2020). Standardized genetic transformation protocol for Chrysanthemum cv. ‘Jinba’ with TERMINAL FLOWER 1 homolog CmTFL1a. Genes (Basel).

[CR23] Hanzawa Y, Money T, Bradley D (2005). A single amino acid converts a repressor to an activator of flowering. Proc Natl Acad Sci U S A.

[CR24] Higuchi Y (2018). Florigen and anti-florigen: flowering regulation in horticultural crops. Breed Sci.

[CR25] Higuchi Y, Hisamatsu T (2015). CsTFL1, a constitutive local repressor of flowering, modulates floral initiation by antagonising florigen complex activity in chrysanthemum. Plant Sci.

[CR26] Higuchi Y, Narumi T, Oda A, Nakano Y, Sumitomo K, Fukai S (2013). The gated induction system of a systemic floral inhibitor, antiflorigen, determines obligate short-day flowering in chrysanthemums. Proc Natl Acad Sci U S A.

[CR27] Ho WW, Weigel D (2014). Structural features determining flower-promoting activity of Arabidopsis FLOWERING LOCUS T. Plant Cell.

[CR28] Hou CJ, Yang CH (2009). Functional analysis of FT and TFL1 orthologs from orchid (Oncidium Gower Ramsey) that regulate the vegetative to reproductive transition. Plant Cell Physiol.

[CR29] Hsu CY, Adams JP, Kim H, No K, Ma C, Strauss SH (2011). FLOWERING LOCUS T duplication coordinates reproductive and vegetative growth in perennial poplar. Proc Natl Acad Sci U S A.

[CR30] Hsu CY, Adams JP, No K, Liang H, Meilan R, Pechanova O (2012). Overexpression of CONSTANS homologs CO1 and CO2 fails to alter normal reproductive onset and fall bud set in woody perennial poplar. PLoS One.

[CR31] Hsu CY, Liu Y, Luthe DS, Yuceer C (2006). Poplar FT2 shortens the juvenile phase and promotes seasonal flowering. Plant Cell.

[CR32] Huang NC, Jane WN, Chen J, Yu TS (2012). Arabidopsis thaliana CENTRORADIALIS homologue (ATC) acts systemically to inhibit floral initiation in Arabidopsis. Plant J.

[CR33] Imaizumi T, Kay SA (2006). Photoperiodic control of flowering: not only by coincidence. Trends Plant Sci.

[CR34] Imaizumi T, Schultz TF, Harmon FG, Ho LA, Kay SA (2005). FKF1 F-box protein mediates cyclic degradation of a repressor of CONSTANS in Arabidopsis. Science.

[CR35] Iwata H, Gaston A, Remay A, Thouroude T, Jeauffre J, Kawamura K (2012). The TFL1 homologue KSN is a regulator of continuous flowering in rose and strawberry. Plant J.

[CR36] Jagadish SV, Bahuguna RN, Djanaguiraman M, Gamuyao R, Prasad PV, Craufurd PQ (2016). Implications of high temperature and elevated CO2 on flowering time in plants. Front Plant Sci.

[CR37] Jiang D, Wang Y, Wang Y, He Y (2008). Repression of FLOWERING LOCUS C and FLOWERING LOCUS T by the Arabidopsis Polycomb repressive complex 2 components. PLoS One.

[CR38] Jiang K, Liberatore KL, Park SJ, Alvarez JP, Lippman ZB (2013). Tomato yield heterosis is triggered by a dosage sensitivity of the florigen pathway that fine-tunes shoot architecture. PLoS Genet.

[CR39] Jiang L, Jiang X, Li Y, Gao Y, Wang S, Ma Y (2022). FT-like paralogs are repressed by an SVP protein during the floral transition in Phalaenopsis orchid. Plant Cell Rep..

[CR40] Kardailsky I, Shukla VK, Ahn JH, Dagenais N, Christensen SK, Nguyen JT (1999). Activation tagging of the floral inducer FT. Science..

[CR41] Karkute SG, Singh AK, Gupta OP, Singh PM, Singh B (2017). CRISPR/Cas9 mediated genome engineering for improvement of horticultural crops. Front Plant Sci.

[CR42] Karlgren A, Gyllenstrand N, Källman T, Sundström JF, Moore D, Lascoux M (2011). Evolution of the PEBP gene family in plants: functional diversification in seed plant evolution. Plant Physiol.

[CR43] Kim W, Park TI, Yoo SJ, Jun AR, Ahn JH (2013). Generation and analysis of a complete mutant set for the Arabidopsis FT/TFL1 family shows specific effects on thermo-sensitive flowering regulation. J Exp Bot.

[CR44] Klintenäs M, Pin PA, Benlloch R, Ingvarsson PK, Nilsson O (2012). Analysis of conifer FLOWERING LOCUS T/TERMINAL FLOWER1-like genes provides evidence for dramatic biochemical evolution in the angiosperm FT lineage. New Phytol.

[CR45] Koskela EA, Mouhu K, Albani MC, Kurokura T, Rantanen M, Sargent DJ (2012). Mutation in TERMINAL FLOWER1 reverses the photoperiodic requirement for flowering in the wild strawberry Fragaria vesca. Plant Physiol.

[CR46] Kumar SV, Lucyshyn D, Jaeger KE, Alós E, Alvey E, Harberd NP (2012). Transcription factor PIF4 controls the thermosensory activation of flowering. Nature..

[CR47] Lazare S, Zaccai M (2016). Flowering pathway is regulated by bulb size in Lilium longiflorum (Easter lily). Plant Biol (Stuttg).

[CR48] Lee JH, Yoo SJ, Park SH, Hwang I, Lee JS, Ahn JH (2007). Role of SVP in the control of flowering time by ambient temperature in Arabidopsis. Genes Dev.

[CR49] Lee R, Baldwin S, Kenel F, McCallum J, Macknight R (2013). FLOWERING LOCUS T genes control onion bulb formation and flowering. Nat Commun.

[CR50] Leeggangers HACF, Rosilio-Brami T, Bigas-Nadal J, Rubin N, van Dijk ADJ, de Caceres N, Gonzalez FF (2018). Tulipa gesneriana and Lilium longiflorum PEBP genes and their putative roles in flowering time control. Plant Cell Physiol..

[CR51] Li DM, L FB, Zhu GF, Sun YB, Liu HL, Liu JW (2014). Molecular characterization and functional analysis of a flowering locus T homolog gene from a Phalaenopsis orchid. Genet Mol Res.

[CR52] Li XF, Jia LY, Xu J, Deng XJ, Wang Y, Zhang W (2013). FT-like NFT1 gene may play a role in flower transition induced by heat accumulation in Narcissus tazetta var. chinensis. Plant Cell Physiol..

[CR53] Lifschitz E, Ayre BG, Eshed Y (2014). Florigen and anti-florigen - a systemic mechanism for coordinating growth and termination in flowering plants. Front Plant Sci.

[CR54] Lifschitz E, Eviatar T, Rozman A, Shalit A, Goldshmidt A, Amsellem Z (2006). The tomato FT ortholog triggers systemic signals that regulate growth and flowering and substitute for diverse environmental stimuli. Proc Natl Acad Sci U S A..

[CR55] Lin MK, Belanger H, Lee YJ, Varkonyi-Gasic E, Taoka K, Miura E (2007). FLOWERING LOCUS T protein may act as the long-distance florigenic signal in the cucurbits. Plant Cell.

[CR56] Liu CH, Fan C (2016). De novo transcriptome assembly of floral buds of pineapple and identification of differentially expressed genes in response to Ethephon induction. Front Plant Sci.

[CR57] Liu CH, Liu Y, Shao XH, Lai D (2018). Comparative analyses of the transcriptome and proteome of Comte de Paris and smooth Cayenne to improve the understanding of Ethephon-induced floral transition in pineapple. Cell Physiol Biochem.

[CR58] Liu H, Yu X, Li K, Klejnot J, Yang H, Lisiero D (2008). Photoexcited CRY2 interacts with CIB1 to regulate transcription and floral initiation in Arabidopsis. Science..

[CR59] Liu L, Li C, Teo ZWN, Zhang B, Yu H (2019). The MCTP-SNARE complex regulates Florigen transport in Arabidopsis. Plant Cell.

[CR60] Liu L, Liu C, Hou X, Xi W, Shen L, Tao Z (2012). FTIP1 is an essential regulator required for florigen transport. PLoS Biol.

[CR61] Liu L, Zhang Y, Yu H (2020). Florigen trafficking integrates photoperiod and temperature signals in Arabidopsis. J Integr Plant Biol.

[CR62] Luo X, Yin M, He Y (2021). Molecular genetic understanding of photoperiodic regulation of flowering time in Arabidopsis and soybean. Int J Mol Sci.

[CR63] Mao Y, Sun J, Cao P, Zhang R, Fu Q, Chen S (2016). Functional analysis of alternative splicing of the FLOWERING LOCUS T orthologous gene in Chrysanthemum morifolium. Hortic Res..

[CR64] Mathieu J, Warthmann N, Küttner F, Schmid M (2007). Export of FT protein from phloem companion cells is sufficient for floral induction in Arabidopsis. Curr Biol.

[CR65] Mathieu J, Yant LJ, Mürdter F, Küttner F, Schmid M (2009). Repression of flowering by the miR172 target SMZ. PLoS Biol.

[CR66] Matsoukas IG, Massiah AJ, Thomas B (2012). Florigenic and antiflorigenic signaling in plants. Plant Cell Physiol..

[CR67] Merini W, Calonje M (2015). PRC1 is taking the lead in PcG repression. Plant J.

[CR68] Michaels SD, Himelblau E, Kim SY, Schomburg FM, Amasino RM (2005). Integration of flowering signals in winter-annual Arabidopsis. Plant Physiol.

[CR69] Mohamed R, Wang CT, Ma C, Shevchenko O, Dye SJ, Puzey JR (2010). Populus CEN/TFL1 regulates first onset of flowering, axillary meristem identity and dormancy release in Populus. Plant J.

[CR70] Molinero-Rosales N, Latorre A, Jamilena M, Lozano R (2004). SINGLE FLOWER TRUSS regulates the transition and maintenance of flowering in tomato. Planta..

[CR71] Moraes TS, Dornelas MC, Martinelli AP (2019). FT/TFL1: Calibrating Plant Architecture. Front Plant Sci..

[CR72] Mouhu K, Kurokura T, Koskela EA, Albert VA, Elomaa P, Hytönen T (2013). The Fragaria vesca homolog of suppressor of overexpression of constans1 represses flowering and promotes vegetative growth. Plant Cell.

[CR73] Mozgova I, Hennig L (2015). The polycomb group protein regulatory network. Annu Rev Plant Biol.

[CR74] Müller-Xing R, Clarenz O, Pokorny L, Goodrich J, Schubert D (2014). Polycomb-group proteins and FLOWERING LOCUS T maintain commitment to flowering in Arabidopsis thaliana. Plant Cell.

[CR75] Nakano Y, Higuchi Y, Yoshida Y, Hisamatsu T (2015). Environmental responses of the FT/TFL1 gene family and their involvement in flower induction in Fragaria × ananassa. J Plant Physiol.

[CR76] Nakano Y, Takase T, Takahashi S, Sumitomo K, Higuchi Y, Hisamatsu T (2019). Chrysanthemum requires short-day repeats for anthesis: gradual CsFTL3 induction through a feedback loop under short-day conditions. Plant Sci.

[CR77] Navarro C, Abelenda JA, Cruz-Oró E, Cuéllar CA, Tamaki S, Silva J, Shimamoto K, Prat S (2011). Control of flowering and storage organ formation in potato by FLOWERING LOCUS T. Nature..

[CR78] Njogu MK, Yang F, Li J, Wang X, Ogweno JO, Chen J (2020). A novel mutation in TFL1 homolog sustaining determinate growth in cucumber (Cucumis sativus L.). Theor Appl Genet.

[CR79] Noy-Porat T, Cohen D, Mathew D, Eshel A, Kamenetsky R, Flaishman MA (2013). Turned on by heat: differential expression of FT and LFY-like genes in Narcissus tazetta during floral transition. J Exp Bot.

[CR80] Oda A, Higuchi Y, Hisamatsu T (2017). Photoperiod-insensitive floral transition in chrysanthemum induced by constitutive expression of chimeric repressor CsLHY-SRDX. Plant Sci.

[CR81] Oda A, Higuchi Y, Hisamatsu T (2020). Constitutive expression of CsGI alters critical night length for flowering by changing the photo-sensitive phase of anti-florigen induction in chrysanthemum. Plant Sci.

[CR82] Oda A, Narumi T, Li T, Kando T, Higuchi Y, Sumitomo K (2012). CsFTL3, a chrysanthemum FLOWERING LOCUS T-like gene, is a key regulator of photoperiodic flowering in chrysanthemums. J Exp Bot.

[CR83] Otagaki S, Ogawa Y, Hibrand-Saint Oyant L, Foucher F, Kawamura K, Horibe T (2015). Genotype of FLOWERING LOCUS T homologue contributes to flowering time differences in wild and cultivated roses. Plant Biol (Stuttg)..

[CR84] Parmar N, Singh KH, Sharma D, Singh L, Kumar P, Nanjundan J (2017). Genetic engineering strategies for biotic and abiotic stress tolerance and quality enhancement in horticultural crops: a comprehensive review. 3. Biotech..

[CR85] Pin PA, Benlloch R, Bonnet D, Wremerth-Weich E, Kraft T, Gielen JJ (2010). An antagonistic pair of FT homologs mediates the control of flowering time in sugar beet. Science..

[CR86] Porri A, Torti S, Romera-Branchat M, Coupland G (2012). Spatially distinct regulatory roles for gibberellins in the promotion of flowering of Arabidopsis under long photoperiods. Development..

[CR87] Putterill J, Varkonyi-Gasic E (2016). FT and florigen long-distance flowering control in plants. Curr Opin Plant Biol.

[CR88] Ramos-Sánchez JM, Triozzi PM, Alique D, Geng F, Gao M, Jaeger KE (2019). LHY2 integrates night-length information to determine timing of poplar photoperiodic growth. Curr Biol.

[CR89] Randoux M, Davière JM, Jeauffre J, Thouroude T, Pierre S, Toualbia Y (2014). RoKSN, a floral repressor, forms protein complexes with RoFD and RoFT to regulate vegetative and reproductive development in rose. New Phytol.

[CR90] Rantanen M, Kurokura T, Jiang P, Mouhu K, Hytönen T (2015). Strawberry homologue of terminal flower1 integrates photoperiod and temperature signals to inhibit flowering. Plant J.

[CR91] Rantanen M, Kurokura T, Mouhu K, Pinho P, Tetri E, Halonen L (2014). Light quality regulates flowering in FvFT1/FvTFL1 dependent manner in the woodland strawberry Fragaria vesca. Front Plant Sci.

[CR92] Sawa M, Kay SA (2011). GIGANTEA directly activates flowering locus T in Arabidopsis thaliana. Proc Natl Acad Sci U S A.

[CR93] Schatlowski N, Creasey K, Goodrich J, Schubert D (2008). Keeping plants in shape: polycomb-group genes and histone methylation. Semin Cell Dev Biol.

[CR94] Serrano-Mislata A, Goslin K, Zheng B, Rae L, Wellmer F, Graciet E (2017). Regulatory interplay between LEAFY, APETALA1/CAULIFLOWER and TERMINAL FLOWER1: new insights into an old relationship. Plant Signal Behav.

[CR95] Shalit A, Rozman A, Goldshmidt A, Alvarez JP, Bowman JL, Eshed Y (2009). The flowering hormone florigen functions as a general systemic regulator of growth and termination. Proc Natl Acad Sci U S A.

[CR96] Shannon S, Meeks-Wagner DR (1991). A mutation in the Arabidopsis TFL1 gene affects inflorescence meristem development. Plant Cell.

[CR97] Song C, Li G, Dai J, Deng H (2021). Genome-wide analysis of PEBP genes in Dendrobium huoshanense: unveiling the antagonistic functions of FT/TFL1 in flowering time. Front Genet.

[CR98] Song J (2020). Map-based cloning and functional analysis of the flowering gene FTL1 in tomato. Master thesis, Chinese Academy of Agricultural Sciences Thesis.

[CR99] Song YH, Shim JS, Kinmonth-Schultz HA, Imaizumi T (2015). Photoperiodic flowering: time measurement mechanisms in leaves. Annu Rev Plant Biol.

[CR100] Soyk S, Müller NA, Park SJ, Schmalenbach I, Jiang K, Hayama R (2017). Variation in the flowering gene SELF PRUNING 5G promotes day-neutrality and early yield in tomato. Nat Genet.

[CR101] Srikanth A, Schmid M (2011). Regulation of flowering time: all roads lead to Rome. Cell Mol Life Sci.

[CR102] Su J, Jiang J, Zhang F, Liu Y, Ding L, Chen S (2019). Current achievements and future prospects in the genetic breeding of chrysanthemum: a review. Hortic Res..

[CR103] Sun J, Cao P, Wang L, Chen S, Chen F, Jiang J (2018). The loss of a single residue from CmFTL3 leads to the failure of florigen to flower. Plant Sci.

[CR104] Sun J, Wang H, Ren L, Chen S, Chen F, Jiang J (2017). CmFTL2 is involved in the photoperiod- and sucrose-mediated control of flowering time in chrysanthemum. Hortic Res..

[CR105] Susila H, JurićS LL, Gawarecka K, Chung KS, Jin S (2021). Florigen sequestration in cellular membranes modulates temperature-responsive flowering. Science..

[CR106] Tang M, Bai X, Wang J, Chen T, Meng X, Deng H (2022). Efficiency of graft-transmitted JcFT for floral induction in woody perennial species of the Jatropha genus depends on transport distance. Tree Physiol.

[CR107] Taoka K, Ohki I, Tsuji H, Kojima C, Shimamoto K (2013). Structure and function of florigen and the receptor complex. Trends Plant Sci.

[CR108] Tsuji H (2017). Molecular function of florigen. Breed Sci.

[CR109] Tsuji H, Taoka K (2014). Florigen signaling Enzymes.

[CR110] Turck F, Roudier F, Farrona S, Martin-Magniette ML, Guillaume E, Buisine N (2007). Arabidopsis TFL2/LHP1 specifically associates with genes marked by trimethylation of histone H3 lysine 27. PLoS Genet.

[CR111] Varkonyi-Gasic E, Moss SMA, Voogd C, Wang T, Putterill J, Hellens RP (2013). Homologs of FT, CEN and FD respond to developmental and environmental signals affecting growth and flowering in the perennial vine kiwifruit. New Phytol.

[CR112] Wang F, Gao Y, Liu Y, Zhang X, Gu X, Ma D (2019). BES1-regulated BEE1 controls photoperiodic flowering downstream of blue light signaling pathway in Arabidopsis. New Phytol.

[CR113] Wang L, Sun J, Ren L, Zhou M, Han X, Ding L (2020). CmBBX8 accelerates flowering by targeting CmFTL1 directly in summer chrysanthemum. Plant Biotechnol J.

[CR114] Wang S, Li H, Li Y, Li Z, Qi J, Lin T (2020). FLOWERING LOCUS T improves cucumber adaptation to higher latitudes. Plant Physiol.

[CR115] Wang Y, Liu L, Song S, Li Y, Shen L, Yu H (2017). DOFT and DOFTIP1 affect reproductive development in the orchid Dendrobium Chao Praya smile. J Exp Bot.

[CR116] Wei Q, Ma C, Xu Y, Wang T, Chen Y, Lu J (2017). Control of chrysanthemum flowering through integration with an aging pathway. Nat Commun.

[CR117] Wellmer F, Riechmann JL (2010). Gene networks controlling the initiation of flower development. Trends Genet.

[CR118] Wen C, Zhao W, Liu W, Yang L, Wang Y, Liu X (2019). CsTFL1 inhibits determinate growth and terminal flower formation through interaction with CsNOT2a in cucumber. Development..

[CR119] Wu YM, Ma YJ, Wang M, Zhou H, Gan ZM, Zeng RF (2022). Mobility of FLOWERING LOCUS T protein as a systemic signal in trifoliate orange and its low accumulation in grafted juvenile scions. Hortic Res.

[CR120] Xi W, Liu C, Hou X, Yu H (2010). MOTHER OF FT AND TFL1 regulates seed germination through a negative feedback loop modulating ABA signaling in Arabidopsis. Plant Cell.

[CR121] Xu F, Rong X, Huang X, Cheng S (2012). Recent advances of flowering locus T gene in higher plants. Int J Mol Sci.

[CR122] Xu J, Hua K, Lang Z (2019). Genome editing for horticultural crop improvement. Hortic Res.

[CR123] Yamaguchi A, Kobayashi Y, Goto K, Abe M, Araki T (2005). TWIN SISTER OF FT (TSF) acts as a floral pathway integrator redundantly with FT. Plant Cell Physiol..

[CR124] Yang LW, Wen XH, Fu JX, Dai SL (2018). ClCRY2 facilitates floral transition in Chrysanthemum lavandulifolium by affecting the transcription of circadian clock-related genes under short-day photoperiods. Hortic Res..

[CR125] Yang Y, Ma C, Xu Y, Wei Q, Imtiaz M, Lan H (2014). A zinc finger protein regulates flowering time and abiotic stress tolerance in Chrysanthemum by modulating gibberellin biosynthesis. Plant Cell.

[CR126] Yoo SJ, Chung KS, Jung SH, Yoo SY, Lee JS, Ahn JH (2010). BROTHER OF FT AND TFL1 (BFT) has TFL1-like activity and functions redundantly with TFL1 in inflorescence meristem development in Arabidopsis. Plant J.

[CR127] Yoo SY, Kardailsky I, Lee JS, Weigel D, Ahn JH (2004). Acceleration of flowering by overexpression of MFT (MOTHER OF FT AND TFL1). Mol Cells.

[CR128] Zhang S, Zhang Y, Li K, Yan M, Zhang J, Yu M (2021). Nitrogen mediates flowering time and nitrogen use efficiency via floral regulators in Rice. Curr Biol.

[CR129] Zhao W, Gu R, Che G, Cheng Z, Zhang X (2018). CsTFL1b may regulate the flowering time and inflorescence architecture in cucumber (Cucumis sativus L.). Biochem Biophys Res Commun.

[CR130] Zhou S, Jiang L, Guan S, Gao Y, Gao Q, Wang G (2018). Expression profiles of five FT-like genes and functional analysis of PhFT-1 in a Phalaenopsis hybrid. Electron J Biotechnol.

[CR131] Zhu Y, Klasfeld S, Jeong CW, Jin R, Goto K, Yamaguchi N (2020). TERMINAL FLOWER 1-FD complex target genes and competition with FLOWERING LOCUS T. Nat Commun.

